# Confining TiO_2_ Nanotubes in PECVD-Enabled Graphene Capsules Toward Ultrafast K-Ion Storage: In Situ TEM/XRD Study and DFT Analysis

**DOI:** 10.1007/s40820-020-00460-y

**Published:** 2020-06-09

**Authors:** Jingsheng Cai, Ran Cai, Zhongti Sun, Xiangguo Wang, Nan Wei, Feng Xu, Yuanlong Shao, Peng Gao, Shixue Dou, Jingyu Sun

**Affiliations:** 1 College of Energy, Soochow Institute for Energy and Materials InnovationS (SIEMIS), Key Laboratory of Advanced Carbon Materials and Wearable Energy Technologies of Jiangsu Province, Soochow University, Suzhou, 215006 Jiangsu People’s Republic of China; 2 SEU-FEI Nano-Pico Center, Key Laboratory of MEMS of Ministry of Education, Southeast University, Nanjing, 210096 People’s Republic of China; 3 Beijing Graphene Institute (BGI), Beijing, 100095 People’s Republic of China; 4 Electron Microscopy Laboratory, International Centre for Quantum Materials, School of Physics, Peking University, Beijing, 100871 People’s Republic of China; 5 Institute for Superconducting and Electronic Materials, University of Wollongong, Wollongong, NSW 2522 Australia

**Keywords:** TiO_2_, Potassium storage, In situ TEM, Plasma-enhanced CVD, Graphene

## Abstract

Titanium dioxide (TiO_2_) has gained burgeoning attention for potassium-ion storage because of its large theoretical capacity, wide availability, and environmental benignity. Nevertheless, the inherently poor conductivity gives rise to its sluggish reaction kinetics and inferior rate capability. Here, we report the direct graphene growth over TiO_2_ nanotubes by virtue of chemical vapor deposition. Such conformal graphene coatings effectively enhance the conductive environment and well accommodate the volume change of TiO_2_ upon potassiation/depotassiation. When paired with an activated carbon cathode, the graphene-armored TiO_2_ nanotubes allow the potassium-ion hybrid capacitor full cells to harvest an energy/power density of 81.2 Wh kg^−1^/3746.6 W kg^−1^. We further employ in situ transmission electron microscopy and *operando* X-ray diffraction to probe the potassium-ion storage behavior. This work offers a viable and versatile solution to the anode design and in situ probing of potassium storage technologies that is readily promising for practical applications.

**Electronic supplementary material:**

The online version of this article (10.1007/s40820-020-00460-y) contains supplementary material, which is available to authorized users.

## Highlights

- G-TiO_2_ was fabricated via direct CVD route by growing few-layered graphene capsules over TiO_2_ nanotubes.
- K-ion hybrid capacitors based on the G-TiO_2_ anode and AC cathode synergized high energy and high power density.

**Electronic supplementary material:**

The online version of this article (10.1007/s40820-020-00460-y) contains supplementary material, which is available to authorized users.

## Graphical abstract

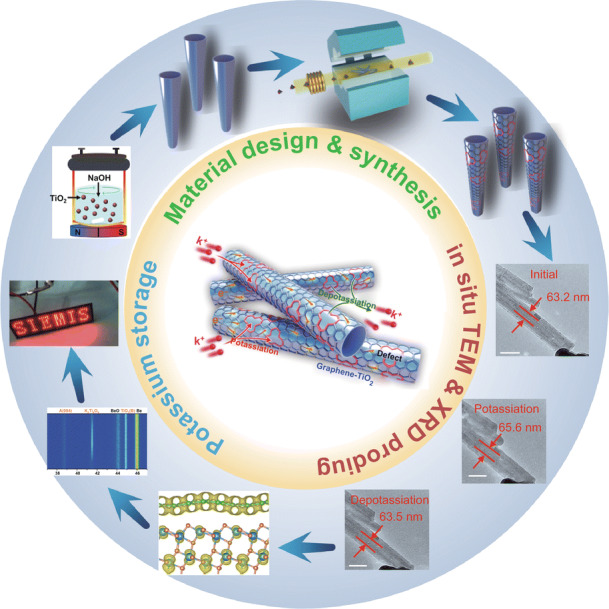

## Introduction

Eco-friendly and sustainable energy storage systems play a vital role in the development of human society [1]. Despite the successful commercialization of lithium-ion batteries, the deficiency and uneven distribution of lithium resources nevertheless render it impractical to meet the ever-growing requirements of large-scale energy storage [2, 3]. Recently, alternative metal-ion batteries (such as sodium and potassium) have stimulated massive attentions, owing to their similar electrochemistry to lithium, earth abundance (2.36, 2.09, and 0.0017 wt% in the earth’s crust for Na, K, and Li, respectively), and cost-effectiveness [4, 5]. In particular, potassium exhibits a lower standard redox potential (− 2.936 V vs. standard hydrogen electrode) than that of Na (− 2.714 V), expecting a higher operating voltage window and an advanced energy density for potassium-ion batteries (KIBs) [6]. However, these merits have been plagued by the sluggish reaction kinetics during the (de)potassiation of large-sized K ions [1.38 Å, higher than Na^+^ (1.02 Å) and Li^+^ (0.76 Å)] at the anode side [7]. As such, nanostructured design of electrode materials with open frameworks and/or topological defects is promising for the construction of high-performance potassium-ion-based energy storage systems, including KIBs and potassium-ion hybrid capacitors (KICs).

Titanium dioxide (TiO_2_) has been probed as a feasible anode candidate in alkali metal-ion batteries because of the high theoretical capacity, broad availability, and environmental benignity [8–11]. Among versatile TiO_2_ nanostructures, one-dimensional TiO_2_ nanotube has gained wide attentions, which provides facile ion transport pathways and ensures adequate electrode–electrolyte contact [12, 13]. However, the intrinsic conductivity of TiO_2_ poses a daunting threat to the rate performance especially under high current densities, thereby resulting in an inferior potassium-ion storage [14]. To tackle this concern, a prevailing approach lies in the synergy of TiO_2_ with conductive media, such as carbonaceous materials, to efficiently boost the conductivity of TiO_2_-based anodes. For instance, the construction of TiO_2_–carbon heterostructure via a wet-chemical method was realized for advanced potassium-ion storage, relying upon a carbon content up to 28.1 wt% [13]. Nevertheless, it remains challenging by far to build a close contact between TiO_2_ and conducting carbon; hence, particle agglomeration and/or volume expansion still occurs during the (de)potassiation process, giving rise to shortened cycle life. The ineffective interface of TiO_2_ and carbon might in addition impede the transport path of K ions to the surface of TiO_2_, thereby handicapping the pseudocapacitive contribution from TiO_2_. The high dosage of carbon would also undermine the energy density of resulting KIBs [15, 16]. Meanwhile, detailed reaction process in terms of (de)potassiation and morphology evolution upon K^+^-ion uptake/release of TiO_2_ anode is still relatively poorly understood.

Herein, we report an in situ synthetic design of graphene-armored TiO_2_ nanotubes (G-TiO_2_ NTs) with pseudocapacitive potassium storage as reliable anode material for KIBs. The as-obtained G-TiO_2_ composite was fabricated via the direct growth of graphene on TiO_2_ NTs with the aid of plasma-enhanced chemical vapor deposition (PECVD) in a facile and scalable fashion. The unique architecture of G-TiO_2_ NTs possesses several major advantages: (1) the robust and intimate contact established between graphene and TiO_2_ affords outstanding electrical conductivity, which aids the capacity utilization of TiO_2_ cores; (2) the PECVD procedure allows the creation of topological defects within graphene overlayers, in turn helping easy permeation of electrolyte and facile intercalation of K ions; and (3) the armored graphene shells enable the effective cushion of volume change during the insertion/extraction of K ions, thereby improving the structural and electrochemical stability. As expected, thus-derived G-TiO_2_ NTs manifest excellent pseudocapacitive potassium storage performance with a high reversible capacity of 332 mAh g^−1^ at 0.05 A g^−1^ and an ultrastable high-rate cyclic stability (a capacity fading of 0.008% per cycle at 5 A g^−1^ for 3000 cycles), outperforming the state-of-the-art Ti-based counterparts. The potassium storage behavior pertaining to the G-TiO_2_ NTs is systematically probed throughout in situ transmission electron microscopy and *operando* X-ray diffraction, in combination with *first*-*principle* calculations. Furthermore, as a proof-of-concept demonstration, a KIC full cell constructed with an activated carbon cathode and a G-TiO_2_ NT anode displays a high output voltage of ~ 3 V and favorable energy density/power density of 81.2 Wh kg^−1^/3747 W kg^−1^, suggesting the potential for practical applications.

## Experimental

### Synthesis of TiO_2_ NTs

Briefly, 0.2 g of commercial TiO_2_ powder (P25) and 30 mL of NaOH solution (10 M) were added into a Teflon-lined autoclave (50 mL). The autoclave was then transferred into an oil bath and heated at 130 °C for 24 h accompanied by a continuous magnetic stirring. After cooling down to room temperature, a white jelly-like suspension was obtained, which was subsequently rinsed with deionized water for several times to reach a pH value of 7. Finally, TiO_2_ NTs were produced after dipping the precipitate in 0.1 M HNO_3_ solution for 24 h to displace sodium ions, followed by annealing in air at 450 °C for 3 h.

### Direct PECVD Production of G-TiO_2_ NTs

Thus-prepared TiO_2_ NTs were served as the growth substrate and evenly placed into a CVD tube furnace. The system was pumped to a base pressure of 0.1 Pa and then purged with highly purified Ar to remove the air. The furnace was afterward heated to 500 °C under an Ar atmosphere. A mixture of Ar (50 sccm) and CH_4_ (10 sccm) was introduced with the presence of plasma (80 W) to trigger the reaction and maintained for 40 min to obtain the final products G-TiO_2_ NTs.

### Characterizations

The morphologies of the samples were determined by scanning electron microscopy (SEM, Hitachi, SU-8010) and transmission electron microscopy (TEM, FEI, Tecnai G2 F20, 80–300 kV) equipped with an energy-dispersive X-ray spectroscopy (EDS). The structures of the samples were characterized by X-ray diffraction (XRD) employing an X-ray diffractometer (D8 Advance, Bruker Inc., 40 kV, 40 mA, a nickel-filtered Cu Kα radiation) and Raman spectroscopy (Horiba Jobin–Yvon, LabRAM HR800). XPS measurements were taken using a Physical Electronics spectrometer (Quantera II, ULVAC-PHI, Inc.) with an Al Kα source (1486.7 eV) to probe the chemical composition. The carbon content of G-TiO_2_ NTs was quantitatively determined with the aid of a thermogravimetry analyzer (METTLER TOLEDO TGA/DSC1). The conductivity of the samples was measured by using a four-probe resistance measuring system (Guangzhou 4-probe Tech Co. Ltd., RTS-4).

### Electrochemical Measurements

As for KIB half-cells, the working electrode slurry contained active materials (bare TiO_2_ NTs or G-TiO_2_ NTs), sodium alginate (Aldrich), and carbon black (Timcal, Switzerland) in Milli-Q water with a weight ratio of 7:1:2 onto a current collector of copper foils (purity 99.999%; thickness 10 µm). Circular electrodes with a diameter of 13 mm were obtained using a punch machine and vacuum-dried at 120 °C for 12 h. The average loading mass of electrode was ca. 1.0 mg cm^−2^. The potassium foil, glass fiber, and a homogenous 0.8 M KPF_6_ solution in ethylene carbonate/dimethyl carbonate (1:1 in volume) were selected as the counter electrode, separator, and electrolyte, respectively. The electrochemical performances were tested on CR2032-type coin cells assembled in an argon-filled glove box with oxygen and water below 0.01 ppm. Galvanostatic discharge/charge cycles were achieved by using the LAND CT2001A battery testing system (Wuhan, China) with a voltage range of 0.01–3.0 V (vs K^+^/K) at room temperature. CV measurements at different scan rates and EIS between 1000 and 0.01 Hz were taken on an Autolab potentiostat (Autolab Instruments, Netherland). As for KIC full cells, CR 2032-type coin cells were constructed with G-TiO_2_ NTs as the anode and PAC as the cathode (weight ratio 1:4). For the fabrication of cathodes, NaOH solution with a certain concentration was used as the pore-forming agent to etch the commercial activated carbon to derive PAC. The PAC electrode was prepared by casting slurries of PAC, polyvinylidene fluoride, and conductive black carbon in N-methyl-2-pyrrolidone (NMP) with a weight ratio of 9:0.5:0.5 onto 15-µm-thick aluminum foils (99.999%). With respect to pre-activation, a KIB was assembled beforehand using G-TiO_2_ NTs as the working electrode, which was charged/discharged for 3 cycles at 0.03 A g^−1^. The gravimetric energy and power densities of the KIC device were calculated by numerically integrating the galvanostatic discharge profiles using Eq. 1:

1 $$ \begin{aligned} E_{\text{mass}} = \int_{{t_{1} }}^{{t_{2} }} {IU/m{\text{d}}t} \hfill \\ P_{\text{mass}} = E_{\text{mass}} /t \hfill \\ \end{aligned} $$

where *I* is the charge/discharge current, *U* is the working voltage, *t*_1_ and *t*_2_ are the start/end-of-discharge time (s), respectively, and *t* corresponds to the discharge time.

### DFT Simulations

First-principle method based on DFT was used to reveal the conductivity of G-TiO_2_ NTs, as implemented by Vienna ab initio simulation package (VASP) [17] software with the projector augmented wave pseudopotential (PAW) [18] to tackle the core and valence electron interactions. The exchange–correlation interactions are handled by generalized gradient approximation (GGA) functional parameterized by Perdew, Burke, and Ernzerhof (PBE) [19]. DFT-D3 method [20] is applied to correct the van der Waals interactions between graphene and TiO_2_ in the G-TiO_2_ composites. The kinetic energy cutoff with the plane wave basis set is 400 eV, and the *k* mesh of 3 × 4×1 and 5 × 8×1 sampling is used to the first Brillouin zone integration for geometric optimization and static calculations, respectively. The convergence criterion of total energy and force per atom are less than 10^−5^ eV and − 0.02 eV Å^−1^, respectively. The model about G-TiO_2_ composite interface is referring to the previous work using 5 × 3 supercell graphene to match 2 × 2 supercell TiO_2_ (101) surface with an angle of ~ 110° [21].

## Results and Discussion

**Fig. 1:**
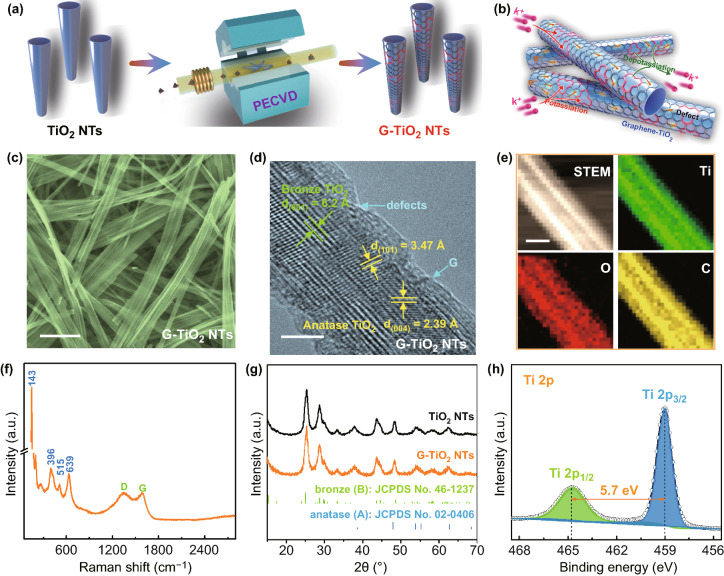
Synthesis and characterization of G-TiO_2_ NTs. **a** Schematic illustration of the direct PECVD synthesis of G-TiO_2_ NTs. **b** A schematic showing electron/K-ion transport within G-TiO_2_ NTs. **c** SEM and **d** HRTEM images of as-prepared G-TiO_2_ NTs. **e** STEM image and corresponding elemental maps of G-TiO_2_ NTs. **f** Raman spectrum of G-TiO_2_ NTs. **g** XRD patterns of bare TiO_2_ NTs and the PECVD-derived G-TiO_2_ NTs. **h** XPS high-resolution Ti 2p spectrum of G-TiO_2_ NTs. Scale bars: **c** 500 nm; **d** 5 nm; **e** 50 nm

As depicted in Fig. 1a, G-TiO_2_ NTs are prepared via a sequential two-step route [22] in a scalable manner. In the first step, commercial TiO_2_ powder (P25) (Fig. S1) and a tailored hydrothermal reaction are employed to produce one-dimensional TiO_2_ tubular nanostructures (Fig. S2). Subsequently, as-obtained TiO_2_ NTs are subject to a direct PECVD process using methane as the carbon precursor (Fig. S3), where defective graphene is in situ formed on the NT surface at a relatively low growth temperature (i.e., 500 °C). The graphene coating generated by such a vapor-phase reaction is of high uniformity, evidenced by the obvious color change from bare white of TiO_2_ powders into dark gray of G-TiO_2_ in macroscopic quantity (Fig. S4). Compared with the routine graphene (such as reduced graphene oxide) [23] incorporation possessing ineffective contact toward active components for KIBs, the conformal caging of defective graphene overlayers can not only facilitate the electron transport and K-ion diffusion, but also confine the TiO_2_ cores to accommodate the volume change that may occur upon charging/discharging (Fig. 1b). This would ultimately be beneficial to improving the rate capability and cycling stability.

Representative SEM image of thus-fabricated TiO_2_ exhibits an interwound nanotube morphology, with over 10 μm in length and 20 nm in average width (Fig. S5). The structure of TiO_2_ can be well maintained after the PECVD process (Fig. 1c), indicative of mild reaction conditions for direct graphene wrapping to derive G-TiO_2_. This is verified by TEM examination, readily showing uniform tubular morphologies of G-TiO_2_ (Fig. S6). High-resolution TEM (HRTEM) image in Fig. 1d reveals that the intact root of graphene onto the TiO_2_ NT via direct CVD technique. It shows the lattice spacings of the (101) and (004) facets of the anatase phase and the (001) facet of the TiO_2_ (B) phase, indicative of a dual-phase configuration, which is promising for the insertion/extraction of the alkali metal ions [12, 24]. Scanning transmission electron microscopy (STEM) image and corresponding elemental mappings (Fig. 1e) show the homogeneous distributions of Ti, O, and C elements, further confirming the uniform caging of graphene in situ to construct G-TiO_2_ composite. Figure 1f displays the Raman spectrum of as-prepared G-TiO_2_ NTs. The conspicuous signals at 143, 396, 515, and 639 cm^−1^ are features of TiO_2_ [25]. It also manifests typical graphene signals, which encompasses a D band (1345 cm^−1^) attributed to the disordered carbon and a G band (1590 cm^−1^) attributed to the *sp*^2^ carbon structure [26]. The *I*_D_/*I*_G_ ratio is greater than 1, implying the existence of ample defects within the direct-PECVD-derived graphene overlayers, which can facilitate the ion diffusion to enhance the reaction kinetics [27]. N_2_ adsorption/desorption measurements suggest that PECVD-derived G-TiO_2_ NTs possesses a specific surface area of 52.0 m^2^ g^−1^ (Fig. S7), which is higher than the pure TiO_2_ NTs (45.1 m^2^ g^−1^). The larger surface area and defect-rich graphene of G-TiO_2_ NTs is beneficial to enriching the electron and ion pathways for advancing energy storage applications [15]. The content of graphene caging in the G-TiO_2_ composite was determined to be < 5 wt% by thermogravimetric analysis, according to the weight loss observed from 100 to 780 °C (Fig. S8). Figure 1g exhibits XRD data of TiO_2_ and G-TiO_2_ NTs. Both samples show mixed phases of TiO_2_, namely bronze (B) (PDF#46-1237) and anatase (A) (PDF#02-0406). Note that bronze (B) TiO_2_ is suggested to be more favorable for the intercalation of alkali metal ions [12], thus expecting an advanced potassium storage. To gain further insights into the chemical constitution of G-TiO_2_ NTs, XPS measurements were taken. The survey XPS data disclose the co-existence of Ti, O, and C elements (Fig. S9a). C 1 s high-resolution spectrum is contributed by a sharp *sp*^2^ C=C peak as well as broad C‒O and C=O peaks, indicating the formation of defective graphene by the PECVD method (Fig. S9b). O 1 s high-resolution spectrum (Fig. S9c) can be deconvoluted into two components, which are attributed to the Ti–O–Ti bonding in TiO_2_ and oxygen-containing functional groups on the surface of graphene (C–O), respectively [28]. Figure 1h presents Ti 2p high-resolution XPS profile. The fitting peaks at 464.7 (Ti 2p_1/2_) and 459.0 eV (Ti 2p_3/2_) displays a binding energy gap of 5.7 eV, well suggesting the survival of Ti^4+^ in G-TiO_2_ NTs experiencing the PECVD process [29–31]. Upon direct graphene growth, the electrical conductivity of hybrids affords marked enhancement based on a sheet resistance mapping result (Fig. S10), showing an electrical conductivity value of 4.15 S m^−1^ [32].

**Fig. 2:**
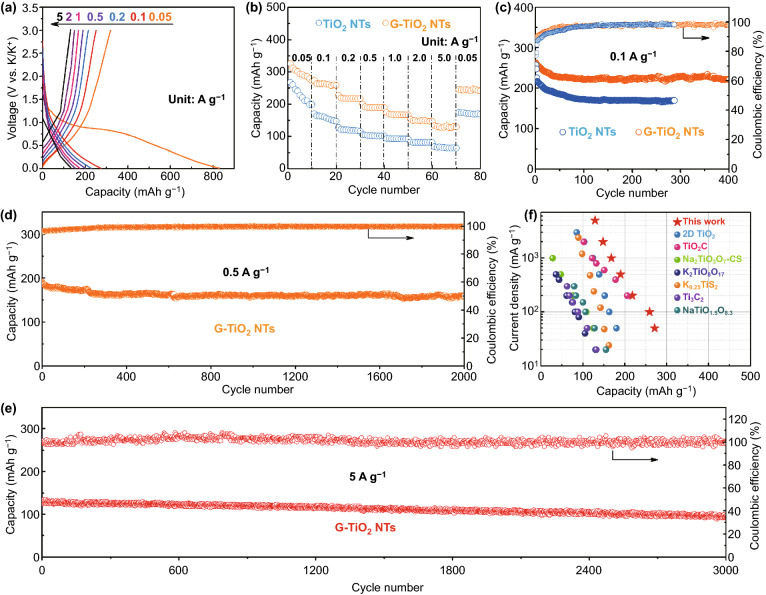
Electrochemical performances of bare TiO_2_ NTs and G-TiO_2_ NTs as anodes in KIBs. **a** Galvanostatic charge–discharge profiles of G-TiO_2_ NTs at various current densities of 0.05–5 A g^−1^. **b** Rate performances of bare TiO_2_ NTs and G-TiO_2_ NTs at various current densities of 0.05–5 A g^−1^. **c** Cycling performances of bare TiO_2_ NTs and G-TiO_2_ NTs at a current density of 0.1 A g^−1^. **d** Cycling performance of G-TiO_2_ NTs at a current density of 0.5 A g^−1^ for 2000 cycles. **e** Cycling performance of G-TiO_2_ NTs at a current density of 5 A g^−1^ for 3000 cycles. **f** Comparison of the rate performances between our G-TiO_2_ NT anode and the state-of-the-art Ti-based KIB anodes

Thus-designed G-TiO_2_ NTs were accordingly explored as anode materials to evaluate the potassium-ion storage performance, with bare TiO_2_ NTs serving as the control. Cyclic voltammetry (CV) of the G-TiO_2_ anode at a scan rate of 0.1 mV s^−1^ for the initial three cycles is shown (Fig. S11). Obviously, the first cathodic scan shows discernible peaks that deal with the decomposition of the electrolyte and the formation of solid electrolyte interphase (SEI) film [33]. The CV profiles overlap quite well at the second and third cycles, indicative of good reversibility. Further, the charge/discharge curves of G-TiO_2_ NTs for the first cycle at different current densities are shown in Fig. 2a. The initial discharge and charge capacities at 0.05 A g^−1^ are 831 and 320 mAh g^−1^, respectively, with a quite low Coulombic efficiency. The large irreversible capacity can also be attributed to the formation of SEI layer during the potassiation process [33]. The following cycles at higher current densities witness stabilized charge/discharge profiles, implying a good reversibility after the initial activation and SEI film formation [16]. Figure 2b compares the rate performances of bare TiO_2_ and G-TiO_2_ NT electrodes at various charge/discharge rates. Augmenting the current density in a step-wise manner from 0.05 to 5 A g^−1^, the G-TiO_2_ NTs deliver a capacity of 271.6, 258.7, 217.3, 189.3, 166.8, 133.4, and 129.2 mAh g^−1^ at the rate of 0.05, 0.1, 0.2, 0.5, 1, 2, and 5 A g^−1^, respectively. When the rate returns to 0.05 A g^−1^, the G-TiO_2_ NTs can still retain a capacity of 245.6 mAh g^−1^, exhibiting strong tolerance for fast potassiation/depotassiation and favorable reversibility. In contrast, the control TiO_2_ NTs without graphene caging and the heat-treated TiO_2_ NTs [34] only harvest a low capacity of 75 and 120 mAh g^−1^ at 2 A g^−1^, respectively. Additionally, they deliver inferior capacities at a higher rate (i.e., 5 A g^−1^) (Fig. S12). The reversible capacity and Coulombic efficiency of the G-TiO_2_ NTs at 0.1 A g^−1^ over 400 cycles are displayed in Fig. 2c. As such, a stable specific capacity of 222 mAh g^−1^ can still be retained after 400 cycles accompanying a high capacity retention of 84.1%, which is evidently superior to that of bare TiO_2_ NT anode (remaining 170 mAh g^−1^ after 300 cycles). As displayed in Fig. 2d, G-TiO_2_ electrode further exhibits an excellent cyclic stability at 0.5 A g^−1^, affording a reversible capacity of 160 mAh g^−1^ after 2000 cycles, which displays a higher capacity and stability as compared to those of other counterparts with different graphene dosages (~ 3% and ~ 8%) (Fig. S13). More significantly, after cycling at a high current density of 5 A g^−1^ for 3000 cycles, the G-TiO_2_ still delivers a capacity of 96 mAh g^−1^ with an extremely low capacity fading of 0.008% per cycle (Fig. 2e), manifesting ultrastable potassium storage capability. To the best of our knowledge, this is the first time that a Ti-based KIB anode with such a stable cycling performance at high rates has been demonstrated. Furthermore, performance comparisons with recent work on Ti-based anodes in KIBs are shown in Fig. 2f [35–39]. Our work shows as one of the best results reported to date (Table S1).

Electrochemical impedance spectroscopy (EIS) was performed to demonstrate a lower charge-transfer resistance and higher K-ion diffusion kinetics of G-TiO_2_ NTs as compared to the control TiO_2_ NTs (Fig. S14), which can be ascribed to the enhanced electronic/ionic conductivity from the intimate graphene/TiO_2_ interface. Galvanostatic intermittent titration technique (GITT) measurements (via discharging at 80 mA g^−1^ for 20 min, followed by an open-circuit relaxation for 30 min) were applied to analyze the K^+^ diffusion coefficient (D_K_^+^) in bare TiO_2_ NTs and G-TiO_2_ NTs based on Eq. 2 [40]:

2 $$ D_{{K^{ + } }} = \frac{{4L^{2} }}{\pi \tau } \left( {\frac{{\vartriangle E_{\text{s}} }}{{\vartriangle E_{\text{t}} }}} \right)^{2} $$

where *t* is the duration time of the current pulse (s), *τ* is the relaxation time (s), ∆*E*_s_ and ∆*E*_t_ are the steady-state potential change (V) by the current pulse and the potential change (V) during the constant pulse after eliminating the *iR* drop, respectively, and *L* is the K^+^ diffusion length (cm). In turn, our results show that the calculated diffusion coefficient (D_K_^+^) of G-TiO_2_ NTs (between 7.59 × 10^−10^ and 3.88 × 10^−11^ cm^2^ s^−1^) is obviously higher than that of TiO_2_ NTs (between 3.74 × 10^−10^ and 4.99 × 10^−12^ cm^2^ s^−1^) upon discharge (Fig. S15), further corroborating advanced K^+^ diffusion kinetics in G-TiO_2_ electrodes. The K^+^ diffusion properties of both anodes were further explored by cyclic voltammetry (CV) at different scan rates of 0.1 to 2.0 mV s^−1^ (Fig. S16). The peak currents display a linear relationship with the square root of scan rates, in this respect, the classical Randles–Sevcik equation (Eq. 3) [41] can be applied to quantify the ion diffusion process:

3 $$ i = \left( {2.69 \times 10^{5} } \right)\cdot\,n^{1.5} \cdot\,A\cdot\,D^{0.5} \cdot\,C_{K} \cdot\,\upsilon^{0.5} $$

where *i*, *n*, *A*, *D*, *C,* and *υ* represent the peak current, charge-transfer number, area of the electrode, K-ion diffusion coefficient, concentration of K ions in the cathode, and the scan rate, respectively. In our case, G-TiO_2_ NTs manifest advanced ion diffusion kinetics than that of TiO_2_ NTs (Fig. S17). All these electrochemical characterizations corroborate the merits of G-TiO_2_ NTs with respect to ultrastable potassium storage performance at high rates and facile electron/K-ion transport.

**Fig. 3:**
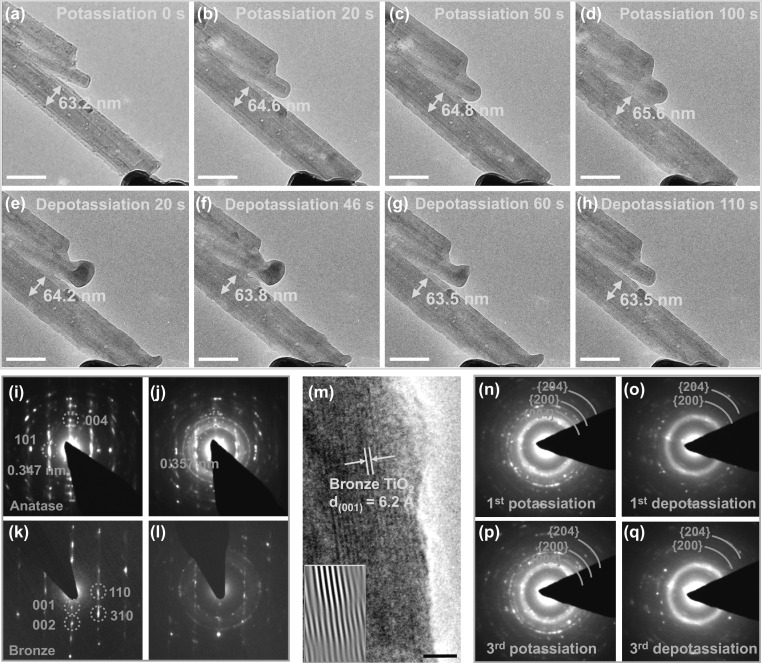
In situ TEM study of G-TiO_2_ NTs upon potassiation/depotassiation. **a**–**d** Time-resolved TEM images showing first electrochemical potassiation process of G-TiO_2_ NTs: **a** A pristine G-TiO_2_ NT. The potassiation was initiated by applying a potential of − 2.0 V to the NTs. **b**, **c** Small expansions in G-TiO_2_ NTs induced by K-ion insertion. **d** Fully potassiated G-TiO_2_ NTs. **e**–**h** The first depotassiation process of G-TiO_2_ NTs, with a potential of +2.0 V applied to extract K ions. **i** Pristine ED pattern of anatase phase. **j** Full potassiation ED pattern of anatase phase. **k** Pristine ED pattern of bronze phase. **l** Full potassiation ED pattern of bronze phase. **m** HRTEM image of potassiated G-TiO_2_ NTs. Inset shows that lattice fringes of (001) plane of bronze phase were slightly distorted because of the K-ion insertion. **n**–**q** ED patterns of the first **n**–**o** and the third **p**, **q** potassiation/depotassiation products to identify the overall reaction mechanism of G-TiO_2_ NTs. Scale bars: **a**–**h** 100 nm; **m** 5 nm

To further probe the durability of G-TiO_2_ NTs with respect to potassium-ion storage, their structural evolutions during (de)potassiation cycles were examined by in situ TEM. The all-solid nanosized KIBs that enabled the real-time observation of in situ electrochemical experiments of G-TiO_2_ NTs were constructed, as depicted in Fig. S18. Figure 3a–d presents the time-lapsed TEM images of different potassiation stages for G-TiO_2_ NTs collected during the first potassiation process (Movie S1). Prior to potassiation, the examined G-TiO_2_ NTs have an original diameter of ~ 63.2 nm. When a potential of − 2 V was applied to the G-TiO_2_ NTs with respect to K electrode, potassium ions began to diffuse along the longitudinal direction starting from the point of contact with the K/K_2_O layer. Such a potassiation process can be visualized by the increased diameter of G-TiO_2_ NTs to 64.6 nm at 20 s (Fig. 3b), resulting in the radial expansion as low as 2.2%. With more potassium insertion, the G-TiO_2_ NTs continue to potassiation and finally acquire radial expansion of 3.7% after full potassiation (Fig. 3c, d), indicating that K storage in G-TiO_2_ NTs almost reaches its maximum capacity. No visible crack and fracture could be observed in the fully potassiated G-TiO_2_ NTs, suggesting a reliable structural evolution. To perform depotassiation, a positive of + 2 V was applied to extract potassium ions from the potassiated G-TiO_2_ NTs. The morphological changes are revealed in Fig. 3e–h and Movie S2. With the extraction of potassium ions, the diameter of the potassiated G-TiO_2_ NTs exhibits a discernible shrinkage from 65.6 to 63.5 nm within 110 s, resulting in a contraction of 3.2%. This implies that the K ions previously inserted could be reversibly extracted, demonstrating the good reversibility of the G-TiO_2_ NTs. For comparison, the potassiation/depotassiation of pure TiO_2_ NTs were also probed by in situ TEM. During the first potassiation (Fig. S19a, b, and Movie S3), TiO_2_ NTs expanded from 110.7 to 114.3 nm within 10 s, leading to a rapid radial expansion of ~ 3.25%. Subsequently, the radial expansion of TiO_2_ NTs increased slightly in next 70 s and eventually reached 22.6% (Fig. S19c, d). As for depotassiation, the diameter of pure TiO_2_ NTs shrinks gradually due to the extraction of K ions (Fig. S19e, f). It is noted that the radial expansion of G-TiO_2_ NTs is significantly lower than that of pure TiO_2_ NTs after the full potassiation. Such conspicuous difference of expansion rates might originate from the mechanical robustness of the graphene coatings [42, 43].

More detailed structural and phase evolution of G-TiO_2_ NTs during the first potassiation were pinpointed by electron diffraction (ED) patterns. Two phases of anatase (A) and bronze (B) of the pristine G-TiO_2_ NTs can be detected in Fig. 3i, k, respectively. Upon potassiation in the anatase phase (Fig. 3j), the phase structure can be well maintained as evidenced by the remaining (101) and (004) diffraction spots, although the interplanar distance of (101) plane slightly expands to 0.357 nm induced by the inserted K ions. Similarly, as with the potassiation in bronze phase (Fig. 3l), the parent diffraction spots become weakened. Figure 3m displays the corresponding HRTEM image of potassiated bronze phase. It is evident that the lattice fringes of (001) plane are slightly distorted as verified by the ED pattern.

To further identify the overall electrochemical reaction mechanism of G-TiO_2_ NTs, cycling performances in anatase phase were investigated. Interestingly, the NTs exhibit multicycle reversible volume expansion/contraction in response to the insertion/extraction of K ions, suggesting the viability of G-TiO_2_ NTs for recyclable KIBs. Accordingly, the ED patterns for the potassiation-depotassiation cycles are acquired in Fig. 3n–q. The first-cycle fully potassiated products are TiO_2_. This result is in accordance with the above observation. Upon full depotassiation, despite the weakened rings, TiO_2_ can still be detected. Moreover, the ED pattern of the third-cycle potassiated/depotassiated products also present the identical features to that of the first-cycle depotassiated products. This indicates that G-TiO_2_ NTs manifest reliable electrochemical cyclic stability.

**Fig. 4:**
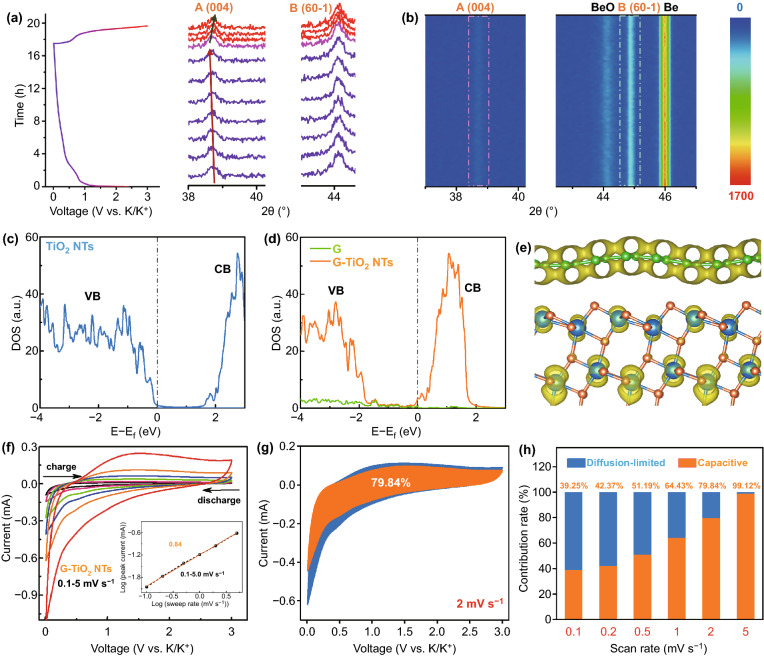
*Operando* XRD, first-principle calculations and kinetics analysis of G-TiO_2_ NTs for potassium storage. **a** The first discharge–charge curve and corresponding *operando* XRD patterns, showing the signal change of key diffractions with K metal serving as the counter electrode. **b** Contour maps for XRD data collected during the first cycle. **c**, **d** Calculated density of states (DOSs) for the c) TiO_2_ and d) G-TiO_2_ system. The black dashed line indicates the Fermi level. **e** Partial charge density around the Fermi level of 0.05 eV by using yellow contour with the iso-surface value of 0.0001 eV/bohr^3^. Ti, C, and O atoms are in blue, green, and orange color, respectively. **f** CV curves of G-TiO_2_ NTs at different scan rates from 0.1 to 5.0 mV s^−1^. Inset: *b*-value determination from the relationship between the peak currents and the scan rates. **g** Separation of the pseudocapacitive contribution (the orange region) for G-TiO_2_ NTs at a CV scan rate of 2 mV s^−1^. **h** Bar chart depicting the percentages of pseudocapacitive contributions at different scan rates from 0.1 to 5 mV s^−1^

*Operando* XRD was further applied as a powerful technique to provide detailed information on the phase evolution of G-TiO_2_ NT and probe the reaction mechanism during the (de)potassiation process. It was implemented on a customized cell with G-TiO_2_ NTs as the working electrode and K metal as the counter electrode. The initial cycle of charge–discharge curve between 0.01 and 3 V and corresponding *operando* XRD patterns are shown in Fig. 4a, with the related contour map in the range of 2*θ* angle plotted in Fig. 4b. In general, the constant signals located at 44.2° and 46.0° are related to the BeO and Be window, respectively [44]. In the meantime, the dominant peak (~ 44.8°) of B-phase TiO_2_ (60-1) shows almost no change in either position or intensity during the entire charge–discharge process, while the A-phase TiO_2_ (004) at ~ 38.7° displays slight shifts toward the low-angle side upon discharge, indicative of the lattice expansion along the A [004] direction upon potassiation, substantiating the occurrence of a K-ion intercalation reaction [45]. In the subsequent charge process, the peak shifts toward high-angle side, implying the dissociation of the intercalated product because of the K-ion extraction. In combination of in situ TEM and *operando* XRD results during the charge/discharge process, potassium-ion storage mechanism of G-TiO_2_ NTs can be described in general by Eqs. 4 and 5:

4 $$ {\text{K}}^{ + } {\text{insertion:}}\quad {\text{TiO}}_{2} + x\left( {{\text{K}}^{ + } + {\text{e}}^{ - } } \right) \to {\text{K}}_{x} {\text{TiO}}_{2} $$

5 $$ {\text{K}}^{ + } {\text{extraction:}}\quad {\text{K}}_{x} {\text{TiO}}_{2} \to {\text{TiO}}_{2} + x{\text{K}}^{ + } + x{\text{e}}^{ - } $$

To elucidate the conductivity enhancement of TiO_2_ NTs after caging graphene (G-TiO_2_ NTs), the first-principle calculation based on DFT was applied to calculate the density of states (DOS) and partial charge density around Fermi level of 0.05 eV (Fig. 4c–e). Initially, G-TiO_2_ model was constructed referring to the previous work by using 5 × 3 graphene to match 2 × 2 anatase TiO_2_ (101) with an angle of ~ 110° (Fig. S20) [21]. DOS calculations indicate that G-TiO_2_ composites display metallic nature as compared to the semiconducting anatase TiO_2_, with the Fermi level shifting up to the conduction band edge of TiO_2_. It means that the charge transfer from graphene toward TiO_2_ surface in the G-TiO_2_ composites. Partial charge density simulations further show that states around Fermi level are mainly contributed by graphene (Fig. 4e), revealing that the markedly enhanced conductivity of G-TiO_2_ NTs is induced by the involvement of directly grown graphene.

Extensive studies have revealed that the exceptional rate performance of anode materials could be related to their high pseudocapacitance. In this respect, CV measurements of G-TiO_2_ NTs at various scan rates from 0.1 to 5.0 mV s^−1^ were performed to interpret this behavior, as displayed in Fig. 4f. Note that similar CV shapes can be attained with a pair of typical redox peaks as the scan rate increases. The charge-storage mechanism can be evaluated according to the relationship between the peak current *i* and the sweep rate *v*: *i *= *aν*^*b*^ (*a* and *b* are adjustable parameters) [46, 47]. The *b*-value can be determined from the slope of log(*ν*)–log(*i*) plot, which lies between 0.5 and 1.0, corresponding to the diffusion-controlled and capacitive-dominant processes, respectively. As with the G-TiO_2_ NT anode, the *b*-value for the anodic peaks is quantified to be 0.84 (Fig. 4f inset), suggesting that the K-ion intercalation mechanism is dominated by pseudocapacitive ion storage behavior.

In further contexts, the capacitive-controlled (*k*_1_*v*) and diffusion-controlled (*k*_2_*v*^1/2^) contributions at given scan rate can be quantitatively determined based on the equation: *i* = *k*_1_*v* + *k*_2_*v*^1/2^ [48, 49]. *i* is the current response associated with the scan rate (*v*), and *k*_1_ and *k*_2_ are constants at a given potential. As displayed in Fig. 4g, a dominant distribution of ca. 79.84% of the total capacity (the light orange shaded area) at 2 mV s^−1^ could be quantified to the pseudocapacitive contribution. Such a contribution is calculated to be higher at higher sweep rates, reaching a maximum value of 99.12% at 5 mV s^−1^ (Fig. 4h). The enhanced pseudocapacitance is indicative of facile electron delivery and K^+^ transport, thereby promoting the rate performance of the G-TiO_2_ NT anode toward ultrastable potassium-ion storage.

**Fig. 5:**
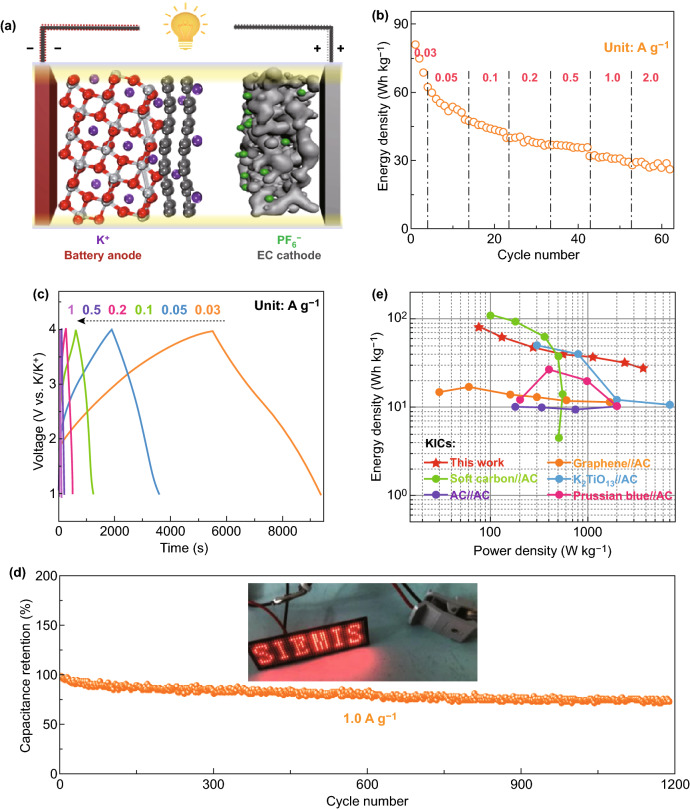
Electrochemical performance of G-TiO_2_ NT-derived KIC full cells. **a** Schematic illustration of G-TiO_2_ NTs//PAC KIC full cell. **b** Rate performance of KIC devices at current densities from 0.03 to 2.0 A g^−1^. **c** Galvanostatic charge/discharge curves of the KIC at different current densities. **d** Capacitance retention of the KIC at 1 A g^−1^ after 1200 cycles. Inset: a photograph showing a “SIEMIS” LED pad powered by G-TiO_2_ NTs//PAC KIC. **e** Ragone plot of G-TiO_2_ NTs//PAC KIC in comparison with other reported KIC systems

Encouraged by the outstanding potassium storage capability of the G-TiO_2_ NTs in half-cells, the KIC full cell is further assembled with the G-TiO_2_ NTs as the anode and porous activated carbon (PAC) as the cathode (G-TiO_2_ NTs//PAC) for proof-of-concept demonstrations, as schematically illustrated in Fig. 5a. During the charge process, K ions partially intercalate into the TiO_2_ NTs via a Faradaic reaction [50] and partially adsorb on the surface of the electrode/defect sites of the graphene through a pseudocapacitive process, while PF_6_^−^ adsorbes on the surface of PAC cathode with a high surface area (~ 1888 m^2^ g^−1^, higher than that of the commercial AC) (Figs. S21 and S22). This process occurs at a voltage range of 1.0–4.0 V to suppress the decomposition of electrolyte at a low potential and side reactions at an exorbitant voltage, generating high-energy and power outputs. Figure 5b exhibits the rate performance of as-constructed KIC full cell, harvesting an energy density of 81.1, 62.5, 47.4, 40.7, 37.6, 33.1, and 28.9 Wh kg^−1^ at a current density of 0.03, 0.05, 0.1, 0.2, 0.5, 1.0, and 2.0 A g^−1^, respectively. The corresponding galvanostatic charge/discharge (GCD) curves are depicted in Fig. 5c, showing typical pseudocapacitive charge-storage features. Furthermore, the G-TiO_2_ NTs//PAC KIC device affords a stable capacity retention over 1200 cycles at 1 A g^−1^ (Fig. 5d). As shown in the inset, a “SIEMIS” light-emitting diode pad can be powered by one individual KIC full cell, indicating its potential application as high-energy/high-power energy storage device. Based on the GCD curves, the energy and power densities of the G-TiO_2_ NTs//PAC KIC can be calculated, which delivers an energy density of 81.2 Wh kg^−1^ at a power density of 75.9 W kg^−1^. It still enables an energy density of 28.1 Wh kg^−1^ at a power output of 3746.6 W kg^−1^. The Ragone plots in Fig. 5e draw a comparison of the energy/power densities between G-TiO_2_ NTs//PAC KIC and the state-of-the-art KICs, manifesting advanced energy/power features of our device as compared to the recently reported systems, such as Graphene//AC [51], AC//AC [52], K_2_TiO_13_//AC [52], Soft carbon//AC [53], and Prussian blue//AC [54].

## Conclusions

In summary, we have developed a direct PECVD strategy to synthesize defective graphene-armored TiO_2_ NTs in a scalable and economic manner. Such in situ coating of graphene shells endows TiO_2_ NTs with fast electron/K-ion transport and favorable structural stability, thereby delivering excellent pseudocapacitive potassium storage performance. Thus-derived KIB cells exhibit a high reversible capacity of 332 mAh g^−1^ at 0.05 A g^−1^ and an unprecedented high-rate cyclic stability at 5 A g^−1^ for 3000 cycles with a capacity fading of 0.008% per cycle. In situ TEM and *operando* XRD, in combination with *first*-*principle* calculations, are employed to systematically probe the potassium storage behavior pertaining to the G-TiO_2_ NTs. Furthermore, the KIC full cell is elaborately constructed, which displays a high output voltage of ~ 3.0 V and high energy density/power density of 81.2 Wh kg^−1^/3747 W kg^−1^. Overall, the unique design of in situ graphene-armored coating to allow marginal volume expansion and high-rate ion intercalation of electrodes opens new avenues for developing next-generation KIB systems and beyond targeting real-life applications.

## Electronic supplementary material

Below is the link to the electronic supplementary material.

Supplementary material 1 (PDF 1341 kb)

Supplementary material 2 (MP4 9737 kb)

Supplementary material 3 (MP4 11447 kb)

Supplementary material 4 (MP4 11455 kb)
