# Supplementary material for: Confining TiO2 Nanotubes in PECVD-Enabled Graphene Capsules Toward Ultrafast K-Ion Storage: In Situ TEM/XRD Study and DFT Analysis
Source: Nanomicro Lett. 2020 Jun 9;12:123. doi: 10.1007/s40820-020-00460-y (PMC7770745; doi:10.1007/s40820-020-00460-y)
Supplement: Supplementary file 1 — Supplementary material 1 (PDF 1341 kb) [file 40820_2020_460_MOESM1_ESM.pdf]

Supporting Information for

Confining TiO_2 Nanotubes in PECVD-Enabled Graphene Capsules toward Ultrafast K-Ion Storage: In Situ TEM/XRD Study and DFT Analysis

Jingsheng Cai[1, ‡], Ran Cai[2, ‡], Zhongti Sun[1, ‡], Xiangguo Wang[1], Nan Wei[1, 3], Feng Xu[2, *], Yuanlong Shao[1, 3], Peng Gao[3, 4, *], Shixue Dou[5], Jingyu Sun[1, 3, *]

[1]College of Energy, Soochow Institute for Energy and Materials InnovationS (SIEMIS), Key Laboratory of Advanced Carbon Materials and Wearable Energy Technologies of Jiangsu Province, Soochow University, Suzhou, Jiangsu 215006, China

[2]SEU-FEI Nano-Pico Center, Key Laboratory of M People's Republic of EMS of Ministry of Education, Southeast University, Nanjing 210096, People's Republic of China

[3]Beijing Graphene Institute (BGI), Beijing 100095, People's Republic of China

[4]Electron Microscopy Laboratory, International Centre for Quantum Materials, School of Physics, Peking University, Beijing 100871, People's Republic of China

[5]Institute for Superconducting and Electronic Materials, University of Wollongong, Wollongong, NSW 2522, Australia

[‡]Jingsheng Cai, Ran Cai, Zhongti Sun contributed equally to this work

*Corresponding authors. E-mail: sunjy86@suda.edu.cn (J. Y. Sun); fxu@seu.edu.cn (F. Xu); p-gao@pku.edu.cn (P. Gao)

Supplementary Figures and Table

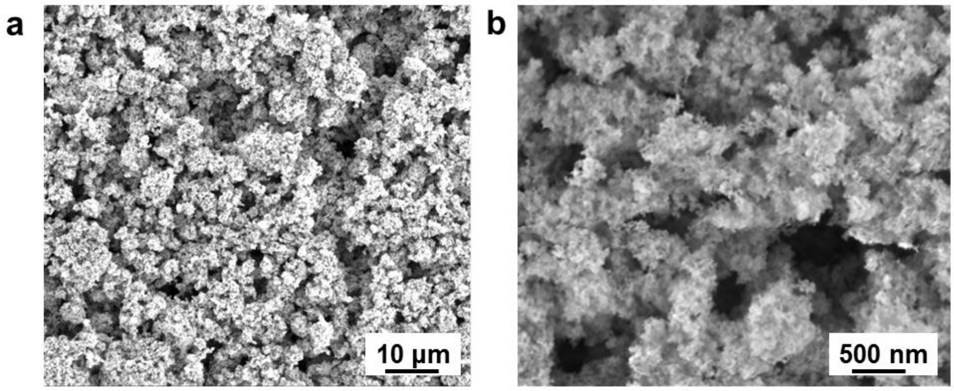

Fig. S1 SEM images of the commercial TiO_2 powder (P25)

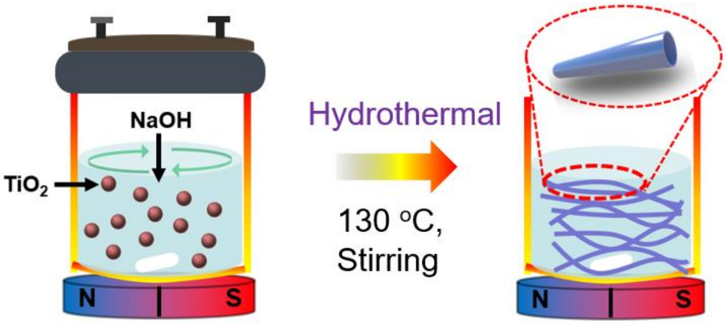

Fig. S2 Schematic illustration of the formation of TiO_2 nanotubes under stirring hydrothermal process at 130 °C for 24 h

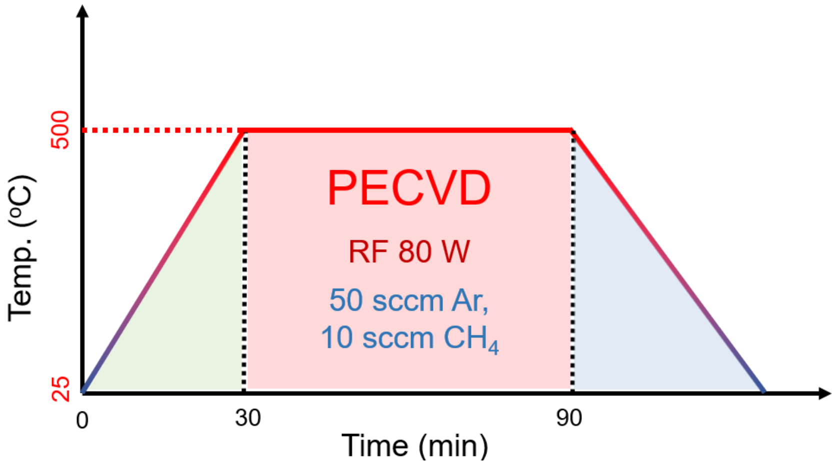

Fig. S3 Experimental procedure of direct PECVD growth of graphene armored coatings on TiO_2 NTs. The direct growth of defective graphene over TiO_2 NTs is inspired by our previous studies pertaining to the direct growth of graphene insulating oxide supports (ACS Nano 2019, 13, 7517; Adv. Mater. 2018, 30, 1800963; ACS Nano 2018, 12, 10240; Nano Res. 2015, 8, 3496; J. Am. Chem. Soc. 2014, 136, 6574)

The formation of uniform coating of graphene on TiO_2 NTs was subject to a well-designed process of direct CVD route, which has been extensively reported by us and others in recent years. Briefly, carbon precursor (CH_4 in this work) was introduced and pyrolyzed by the plasma into various types of carbon fragments prior to reaching the reaction zone. At the surface of TiO_2 NTs, the carbon fragments managed to nucleate, in this regard, the uniform distribution of gas flow (in a low-pressure condition) would aid the uniform nucleation of graphitic carbon. As such, graphene starts to form by means of surface diffusion of carbon, thereby leading to the uniform coating of graphene sheets on TiO_2 NTs.

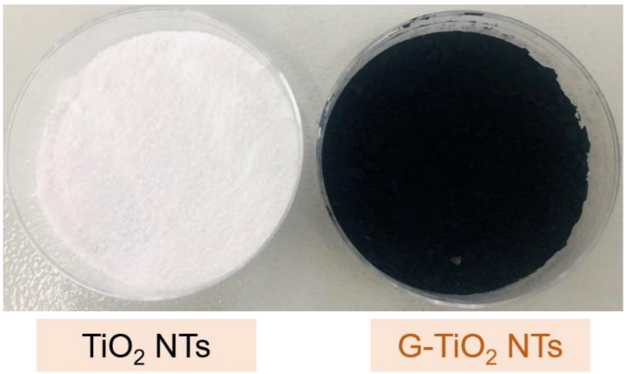

Fig. S4 Digital photographs of the white-colored TiO_2 NTs (left) and the dark-colored PECVD-derived G-TiO_2 NTs (right)

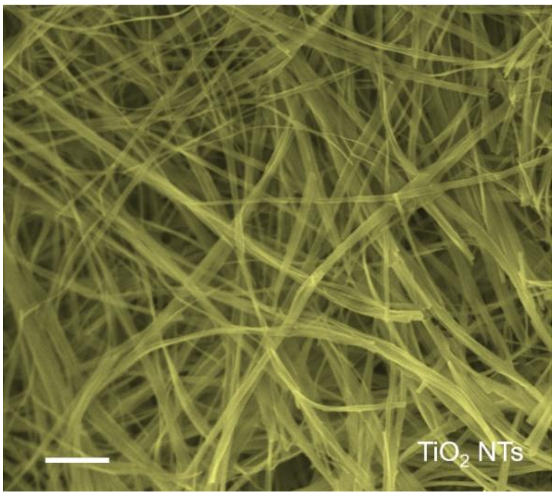

Fig. S5 SEM image of thus-fabricated TiO_2 exhibiting an interwound nanotube morphology. Scale bar: 500 nm

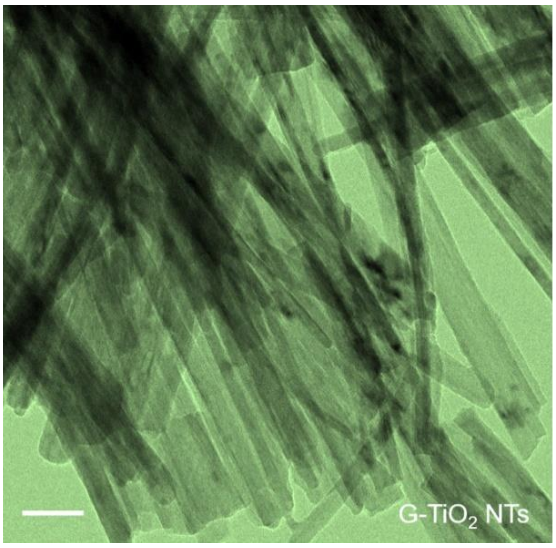

Fig. S6 TEM image of the uniform nanotubular morphologies of G-TiO_2. Scale bar: 200 nm

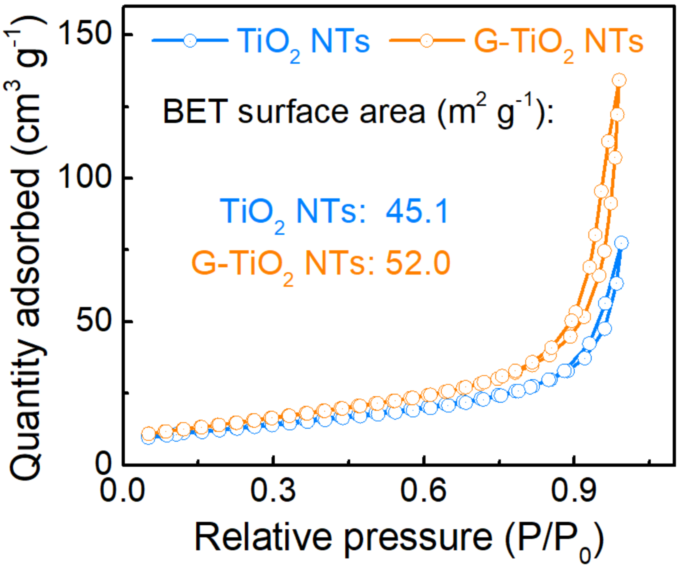

Fig. S7 N_2 adsorption/desorption isotherms of TiO_2 NTs and G-TiO_2 NTs

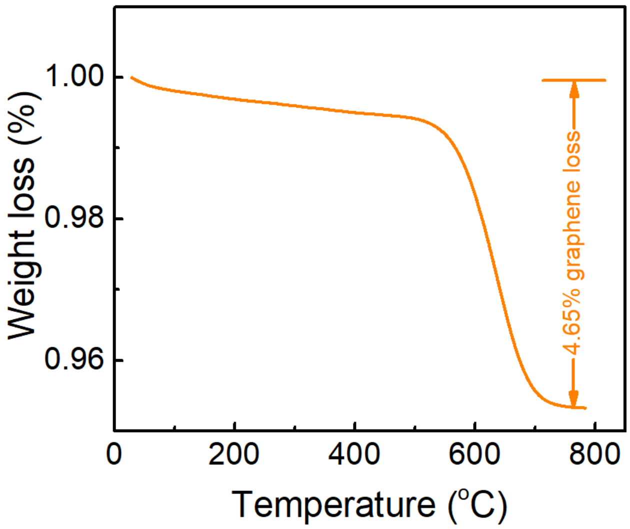

Fig. S8 TG curve of G-TiO_2 NTs, exhibiting a low content of graphene (< 5 wt%)

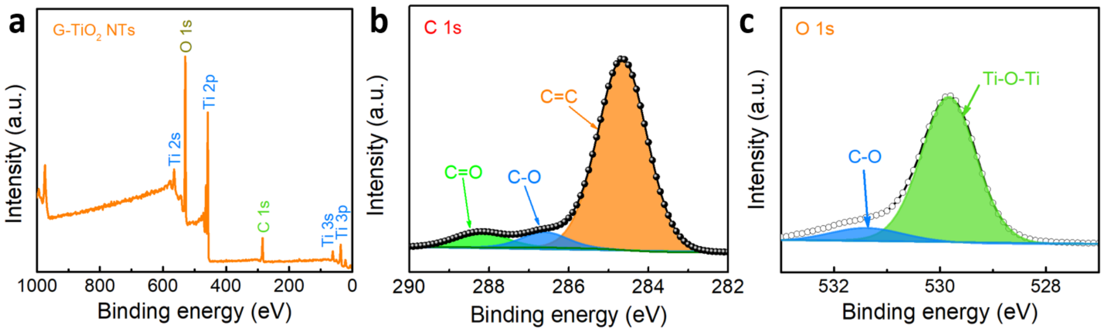

Fig. S9 XPS spectra of the as-prepared G-TiO_2 NTs. **a**) Survey spectrum, **b**) C 1s and **c**) O1s high-resolution spectrum

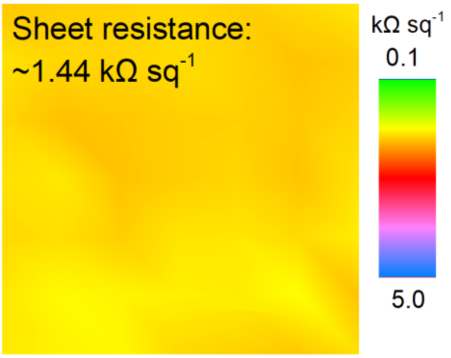

Fig. S10 Sheet resistance mapping of the PECVD-derived G-TiO_2 NT films

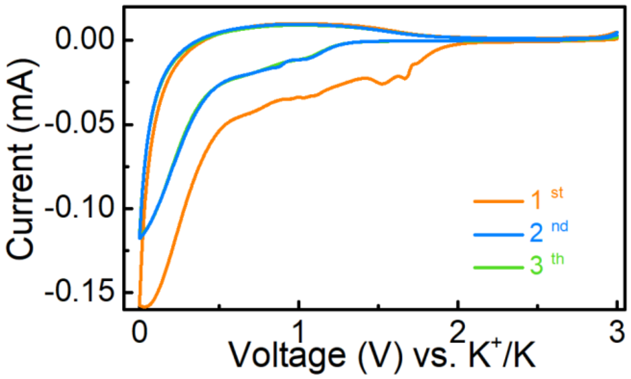

Fig. S11 The first three CV curves of G-TiO_2 electrode at a scan rate of 0.1 mV s^{-1}

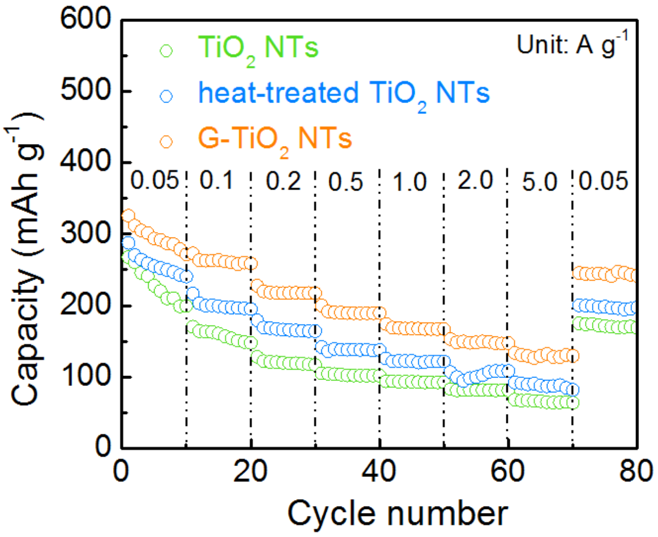

Fig. S12 Rate performances of bare TiO_2 NTs, heat-treated TiO_2 NTs and G-TiO_2 NTs at various current densities of 0.05–5 A g^{-1}

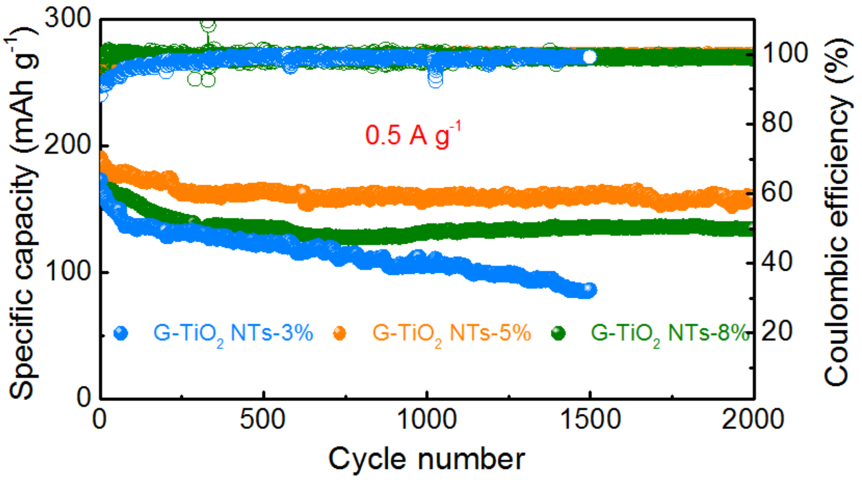

Fig. S13 Cycling performance of G-TiO_2 electrodes with different dosages of graphene (~3%, ~5%, and ~8%) at 0.5 A g^{-1}

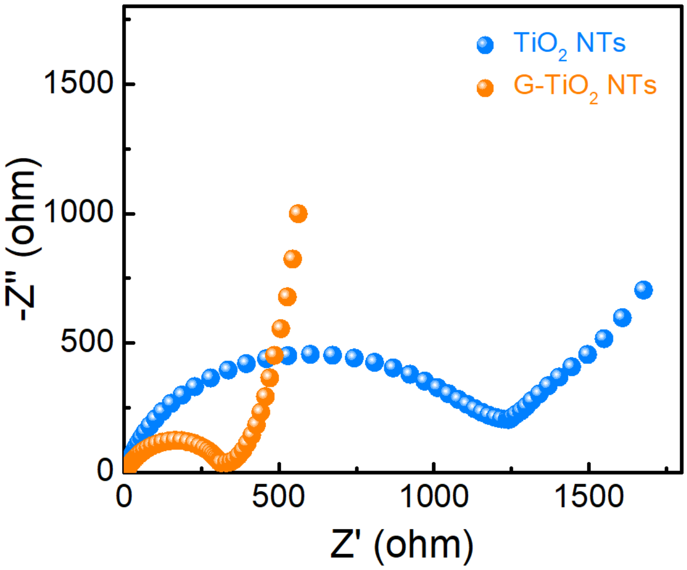

Fig. S14 EIS curves of the TiO_2 NT and G-TiO_2 NT electrodes

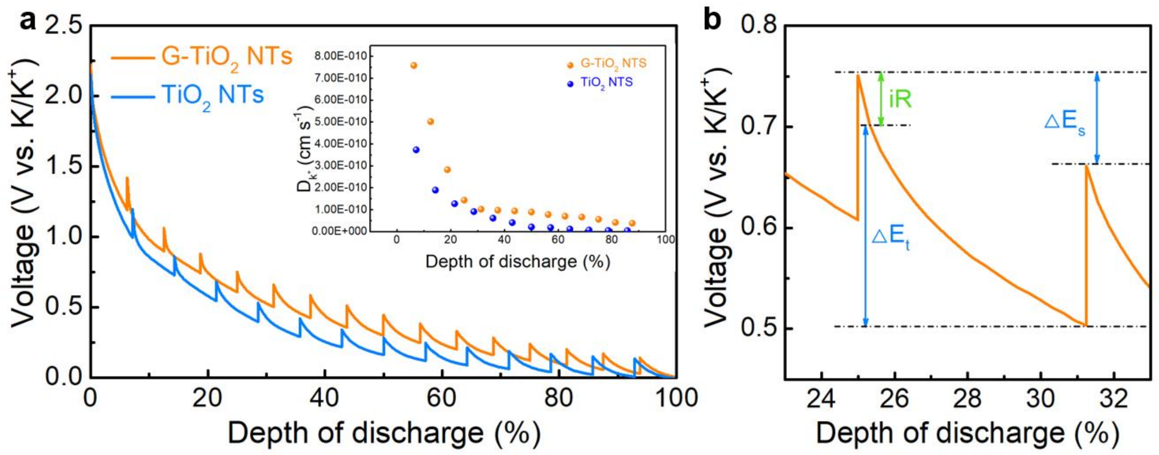

Fig. S15 a) GITT profiles of bare TiO_2 NTs and G-TiO_2 NTs during the discharge process, with the inset showing the corresponding K^+ diffusion coefficients. **b**) A single GITT curve

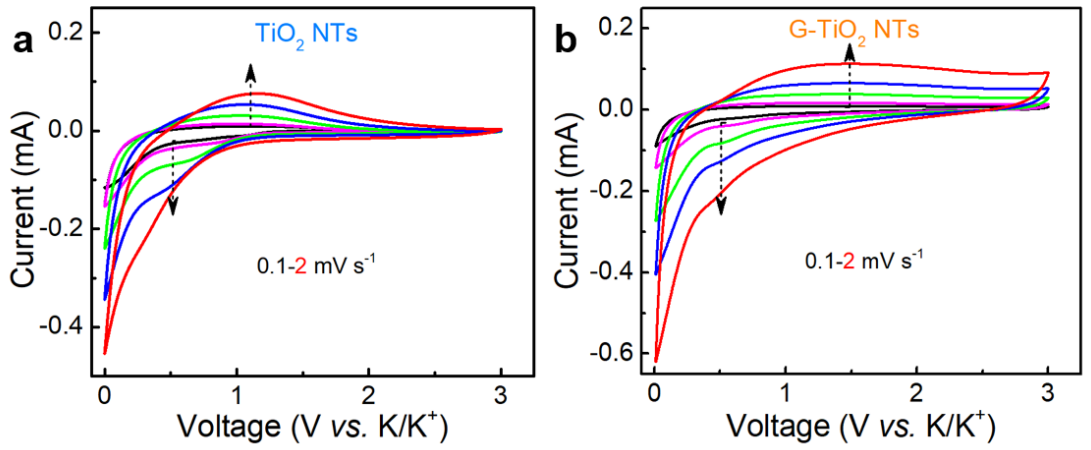

Fig. S16 CV curves of **a**) TiO_2 NT and **b**) G-TiO_2 NT electrodes at different scan rates in range of 0.1−2.0 mV s^{-1}

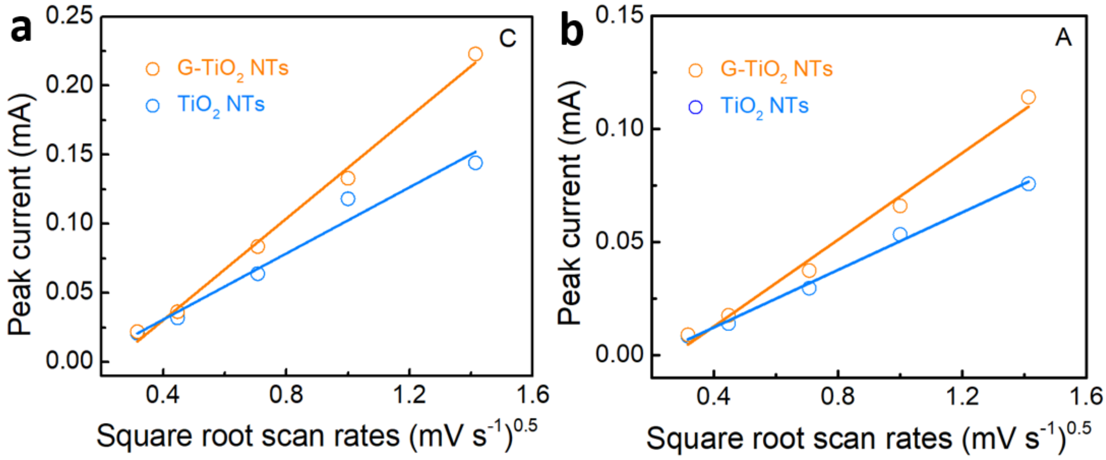

Fig. S17 Reaction kinetics with respect to the K^+-ion diffusion properties of TiO_2 NTs and G-TiO_2 NTs at different scan rates. **a**) Aathodic and **b**) anodic peaks

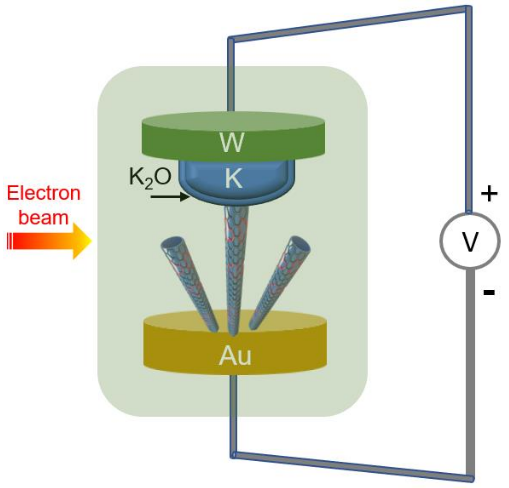

Fig. S18 Schematic illustration of the electrochemical cell for *in-situ* TEM measurement

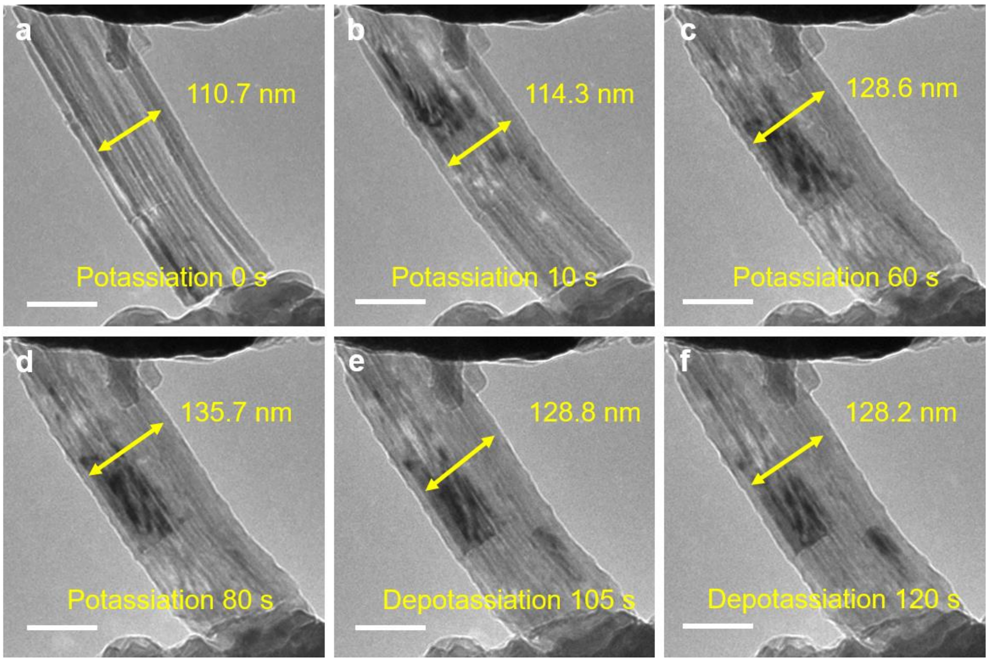

Fig. S19 a-d) Time-resolved TEM images showing the first electrochemical potassiation process of TiO_2 NTs. **e-f**) The first depotassiation process of TiO_2 NTs. A potential of +2.0 V was applied to extract K^+. Scale bars, 100 nm

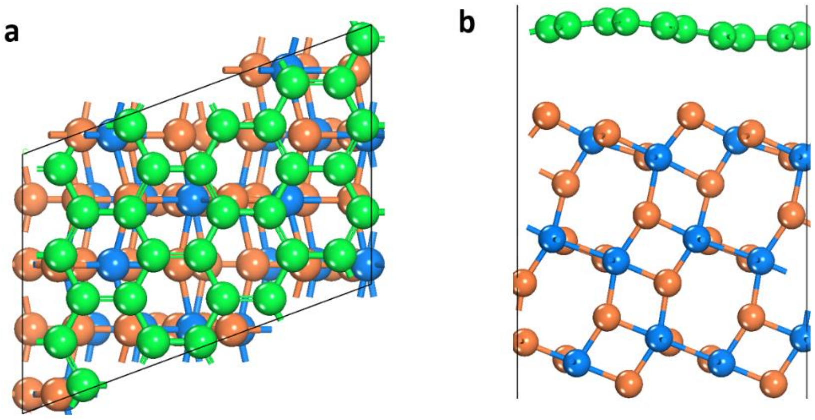

Fig. S20 Calculated structure model of G-TiO_2 system with **a**) top and **b**) side view. Ti, C, and O atoms are in blue, green and orange color

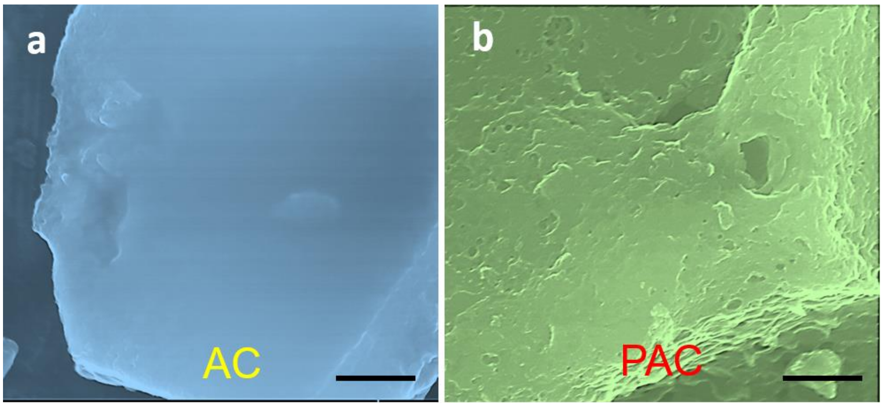

Fig. S21 SEM images of **a**) the AC and **b**) the PAC. Scale bars, 500 nm

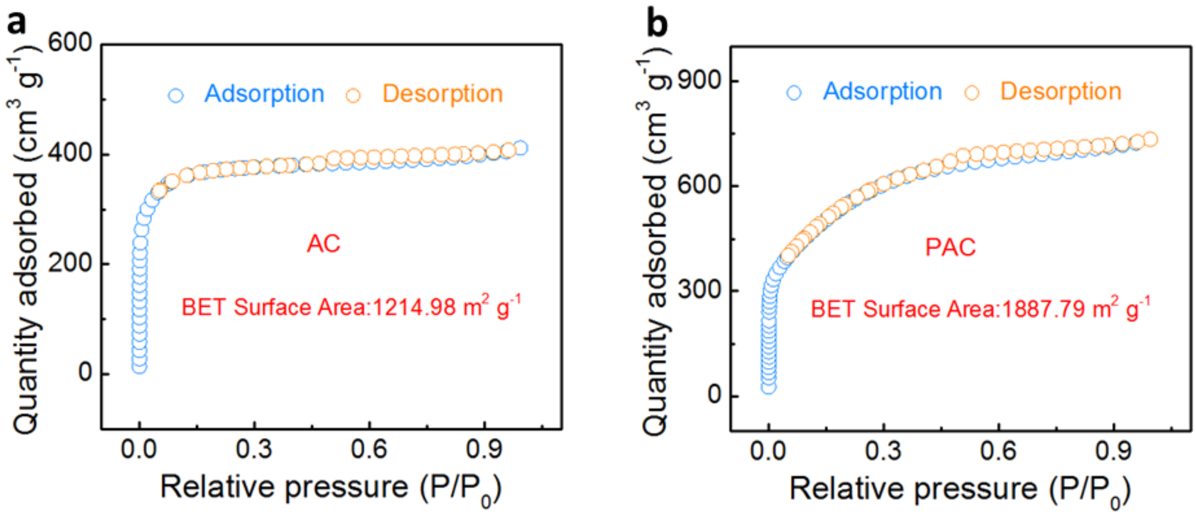

Fig. S22 N_2 adsorption/desorption isotherms of the **a**) AC and **b**) PAC. The derived BET surface area value is displayed

Table S1 Comparison of potassium storage performances based on titanium-based anodes between this work and other reported studies

**Materials**	**Morphology**	**Current density (A g^{-1})**	**Cycles**	**Initial capacity (mAh g^{-1})**	**Capacity decay (%)**	**Ref. (Year)**
G-TiO_2	nanotubes	0.1	400	263.9	15.9	This work
		0.5	2000	195.1	18	
		5.0	3000	129	25.5	
H-TiO_2-C	nanotubes	0.5	1200	163.4	18.7	S1(2019)
$K_2Ti_6O_{13}$	microscaffold	0.5	1000	70.3	16.0	S2(2018)
Ti_3C_2	nanoribbons	0.2	500	~95	-	S3(2017)
$K_2Ti_4O_9$	nanoribbons	0.2	900	~95	49	S4(2017)
TiO_xN_y/C	nanoparticles	0.2	1250	~150	-	S5(2019)
$K_{0.8}[Ti_{1.73}Li_{0.27}]O_4$	nanosheets	1.0	1000	124	<30	S6(2019)
$Ca_{0.5}Ti_2(PO_4)_3$@C	microspheres	1.0	1000	~168	25.4	S7(2018)

Supplementary References

[S1] Y.P. Li, C.H. Yang, F.H. Zheng, Q.C. Pan, Y.Z. Liu et al., Design of TiO_2eC hierarchical tubular heterostructures for high performance potassium ion batteries. Nano Energy **59**, 582−590 (2019). https://doi.org/10.1016/j.nanoen.2019.03.002

[S2] S.Y. Dong, Z.F. Li, Z.Y. Xing, X.Y. Wu, X.L. Ji, X.G. Zhang, Novel potassium-ion hybrid capacitor based on an anode of $K_2Ti_6O_{13}$ microscaffolds. ACS Appl. Mater. Interfaces **10**(18), 15542−15547 (2018). https://doi.org/10.1021/acsami.7b15314

[S3] P.C. Lian, Y.F. Dong, Z.S. Wu, S.H. Zheng, X.H. Wang et al., Alkalized Ti_3C_2 MXene nanoribbons with expanded interlayer spacing for high-capacity sodium and potassium ion batteries. Nano Energy **40**, 1−8 (2017). https://doi.org/10.1016/j.nanoen.2017.08.002

[S4] Y.F. Dong, Z.S. Wu, S.H. Zheng, X.H. Wang, J.Q. Qin et al., Ti_3C_2 MXene-derived sodium/potassium titanate nanoribbons for high-performance sodium/potassium ion batteries with enhanced capacities. ACS Nano **11**(15), 4792−4800 (2017). https://doi.org/10.1021/acsnano.7b01165

[S5] M.L. Tao, G.Y. Du, Y.Q. Zhang, W. Gao, D.Y. Liu et al., TiO_xN_y nanoparticles/C composites derived from MXene as anode material for potassium-ion batteries. Chem. Eng. J 369(1), 828−833 (2019). https://doi.org/10.1016/j.cej.2019.03.144

[S6] J.L. Yang, X. Xiao, W.B. Gong, L. Zhao, G.H. Li et al., Size-independent fast ion intercalation in two-dimensional titania nanosheets for alkali-metal-ion batteries. Angew. Chem. Int. Ed. **58**(26), 8740−8745 (2019). https://doi.org/10.1002/anie.201902478

[S7] Z.Y. Zhang, M.L. Li, Y. Gao, Z.X. Wei, M.N. Zhang et al., Fast potassium storage in hierarchical $Ca_{0.5}Ti_2(PO_4)_3$@C microspheres enabling high-performance potassium-ion capacitors. Adv. Funct. Mater. **28**(36), 1802684 (2018). https://doi.org/10.1002/adfm.201802684
